# Working Memory and Attention – Response to Commentaries

**DOI:** 10.5334/joc.79

**Published:** 2019-08-08

**Authors:** Klaus Oberauer

**Affiliations:** 1 University of Zurich, CH

**Keywords:** Attention, Working memory, Memory

## Abstract

This is a brief reply to the commentaries by Adam and deBettencourt (2019); Allen (2019); Kiyonaga (2019); Schneider (2019); and Van der Stigchel and Olivers (2019), focusing on four topics: (1) I defend the idea that attention need not be characterized as a limited resource. (2) I explain how I conceptualize the role of WM in cognitive control, and how recruitment of sensory processing networks contributes to control but not maintenance. (3) I discuss different ways in which information can be selectively prioritized during or after being encoded into WM, and the different consequences of these processes. (4) I argue that sustained attention to a task can be understood as the mind’s ability to prioritize that task over task-unrelated representations.

I am grateful for the five commentaries on my target article (Oberauer, 2019) because they underscore the diversity of perspectives on the relation of working memory (WM) and attention, and emphasize a few aspects that I had neglected in the target article. I find myself in agreement with most of the comments. In this reply I focus on what I believe to be the most important points for future discussion.

## Attention as a Resource

A common theme of several commentaries is the assumption that attention is a limited resource. For instance, Kiyonaga (2019) argues that attention “is capacity limited and therefore must be selective”, and Allen (2019) describes attention as a limited resource that causes a capacity limit, which “underlines the importance of ‘perceptual’ selection”. I think we should also consider the reverse direction of causality (Neumann, 1987): Attention is the mind’s mechanism for selective prioritization, and the apparent capacity limit arises from this function: As the target of attention is prioritized for processing, competing representations are suppressed to increase the signal-to-noise ratio of selection. As a consequence, it becomes difficult to attend to several distinct representational units at the same time because prioritizing each of them entails suppression of the others.

Allen (2019) argues that the vulnerability of WM performance to dual-task costs demonstrates the involvement of an executive-attention resource in WM. The explanation of dual-task costs as arising from resource sharing is appealing, but there are a few aspects of the dual-task costs suffered by WM tasks that render this explanation questionable. First, dual-task costs are asymmetric between content domains: Visual-spatial WM contents are vulnerable to the concurrent maintenance or processing of verbal information but verbal WM contents are less vulnerable to concurrent maintenance or processing of visual-spatial contents (Morey, 2018; Morey, Morey, van der Reijden, & Holweg, 2013). This asymmetry is difficult to explain by resource sharing: If verbal and visual-spatial representations compete for the same resource, then both should suffer dual-task costs; if they don’t, then none of them should. Second, as I mentioned in the target article, dual-task costs between WM maintenance and a processing demand diminish and often disappear when memory encoding and processing task are temporally separated by a few seconds. This observation rules out the idea that WM maintenance and processing compete for the same resource.

One example for the latter observation is the study of Kiyonaga, Dowd, and Egner (2017): Participants held faces or houses in WM and did a visual-search task (searching for vertical objects) during the retention interval. The difficulty of both task components was varied. Search difficulty had no effect on WM performance, and WM load had no effect on search performance, perhaps because the search task commenced only 2.5 to 5 s after WM encoding. Decoding of the WM content category (face vs. house) from brain areas known to be involved in perceptual processing of these stimuli dropped to chance during the visual-search task. Yet, the WM category could still be decoded from other areas in the ventral stream even during visual search. Kiyonaga (2019) argues that this study shows how competition for resources can be masked by control processes – in this case, processes that transfer WM representations from perceptual-processing areas to other brain areas when visual attention is engaged by a competing demand in the retention interval. I agree that this study attests to the flexibility of WM maintenance: Information about visual stimuli such as faces and houses can be kept active in the relevant perceptual-processing areas, but WM maintenance does not depend on that because the same information is also represented elsewhere throughout the retention interval. At the same time, I don’t see why we need the concept of a shared resource to explain these findings. We don’t need to assume that face and house information disappeared from perceptual areas because a limited resource was taken away from them. An alternative interpretation is that they disappeared because keeping them would be tantamount to using them as search templates that guide perceptual attention (Van der Stigchel & Olivers, 2019), and this would disrupt the search for vertical objects. What looks like competition for a shared resource could simply be a side effect of the fact that attention needs to be selective to fulfil its function.

## WM and Cognitive Control

A second theme touched upon by several commentaries is the role of WM in controlling perceptual attention and action. Schneider (2019) emphasizes that WM is needed for holding bindings between stimulus categories and response categories, especially when they are newly instructed so that they could not have been acquired in long-term memory. Such representations of new task sets in WM are needed not only in task-switching situations, but every time a person wants to implement a new relation between a perceived situation and an action (Meiran, Liefooghe, & De Houwer, 2017).

This idea fits well into the general hypothesis of “attention as an emergent property” proposed by Van der Stigchel and Olivers (2019): Activation of a goal (e.g., to follow the experimenter’s instructions) entails selective activation of representations relevant for that goal, including the instructed mappings between stimulus and response categories (e.g., “if you see a cat, press left; if you see a dog, press right”), and the relevant perceptual and motor representations (e.g., visual images of typical cats and dogs, and motor programs for pressing the left and the right key). Van der Stigchel and Olivers argue that activation of task-relevant perceptual and motor representations agrees well with the sensory-recruitment hypothesis, according to which maintenance of perceptual information in WM relies on the same neural networks that are involved in perceptual processing of that information.

In my view, there is no strong conceptual relation between the attention-as-emergent-property hypothesis and the sensory-recruitment hypothesis. It is probably true that the activation of a goal leads to activation of goal-relevant representations associated to it, including sensory representations, but that does not mean that sensory representations are causally relevant for maintenance of information in WM. They probably are not, because maintenance does not appear to suffer when information in sensory areas is erased by competing stimuli (Bettencourt & Xu, 2016; Kiyonaga et al., 2017). An alternative interpretation of the findings showing information about WM contents in sensory brain areas is that maintenance relies on more abstract representations, which are back-projected into sensory areas. Through this back-projection WM contents could bias perceptual processing. In this interpretation, sensory processing areas are recruited not for maintenance of information in WM but for enabling WM contents to influence perceptual attention. I believe that this idea largely agrees with the position of Van der Stigchel and Olivers, except that I see a place for memory representations that are not currently in the focus of attention, and hence are not maintained in sensory areas, and yet are in WM, rather than being outsourced into long-term memory. If it were easy to outsource all currently unattended WM contents into long-term memory, it would be mysterious why the capacity of WM is so stubbornly limited.

## Attention Controlling the Contents of WM

Allen (2019) describes some of the ways in which attention governs selective encoding of information into WM. Access to WM is controlled by a filter that can implement a conjunction of spatial, temporal, and feature-based criteria for admission (e.g., encoding only the red stimuli on the left of the screen, or only the first four stimuli in a sequence). This filter is imperfect, so that information matching some but not all criteria can slip through – for instance an irrelevant suffix following a set of memory items when the suffix belongs to the same category as the memory items (Allen, Castellà, Ueno, Hitch, & Baddeley, 2015). Kiyonaga (2019) makes a similar observation.

People can also follow instructions to give certain list items priority. Whereas people’s ability to filter out irrelevant items is unaffected by a secondary task, their ability to selectively prioritize some of the memory items is impaired by a secondary task, suggesting that the two processes differ qualitatively (Hu, Allen, Baddeley, & Hitch, 2016). At first glance this dissociation is puzzling because prioritization can be understood as a more lenient form of selective attention, encoding the prioritized items more strongly without completely neglecting the not-prioritized ones. The difference could be explained as follows: Filtering out an irrelevant stimulus is like filtering out distractors in visual search: Attention is guided to stimuli that match the target template, and the more dissimilar stimuli are to that template the less likely they are to be attended, and by implication, encoded into WM. In the suffix paradigm, the target template is constant across trials (“the first four color-shape combinations”) and therefore does not have to be maintained in WM; constant search templates can guide perceptual attention directly from LTM (Woodman, Carlisle, & Reinhart, 2013). Therefore, we should not expect filtering to be impaired by a secondary task. Selectively prioritizing some items could be done by assigning these items more time for encoding, and perhaps post-encoding processing (e.g., short-term consolidation, refreshing, or elaboration) (Oberauer & Lewandowsky, in press). This requires that there is free time for such additional processing, of which there is less when a secondary task is to be carried out concurrently. Therefore, we should expect prioritization to be less effective under dual-task demands, and more generally under conditions with little free time.

Individual items in a memory set can also be prioritized after encoding of an entire memory set when attention is directed to these items during the retention interval by a retro-cue (Souza & Oberauer, 2016). Whereas items prioritized during encoding are made more vulnerable to interference by an irrelevant suffix (Allen, 2019; Allen & Ueno, 2018), items prioritized during the retention interval in response to a retro-cue are made less vulnerable to interference by subsequent visual stimuli (Souza, Rerko, & Oberauer, 2016; van Moorselaar, Gunseli, Theeuwes, & Olivers, 2015). Future research will hopefully find out why these two variants of prioritization have opposing consequences for the prioritized contents’ vulnerability to perceptual interference.

## Working Memory and Sustained Attention

Adam and deBettencourt (2019) describe the covariation of sustained attention and performance in a visual WM task. I am grateful for this addition because I have neglected sustained attention in my review. It is tempting to interpret the waxing and waning of sustained attention as the reflection of an attentional resource that fluctuates over time (deBettencourt, Keene, Awh, & Vogel, 2019), but again, there is an alternative interpretation in terms of selective attention that does not require the concept of a resource: Sustained attention can be characterized as sustained prioritization of task-relevant over task-irrelevant representations. In this view, fluctuations of sustained attention over time reflect the trajectory of “mind wandering”: Sometimes selective attention is directed more towards the experimental task, and on other occasions it is directed more to task-unrelated thoughts. When attention is directed to task-unrelated thoughts, these thoughts are likely to interfere with the experimental task, impairing performance. In line with this interpretation, earlier work has shown that performance in the same visual WM task used by deBettencourt et al. (2019) also correlates with people’s self-report on whether they had been thinking of the WM task or of task-unrelated issues (Adam & Vogel, 2017).

An important question for future research is whether fluctuations of sustained attention are specifically related to WM, or whether they affect performance in a broad class of cognitive activities of which doing a WM task just happens to be an instance. Adam and deBettencourt (2019) highlighted the great potential of experimental techniques to measure the covariation of performance indicators over time. These covariations could become a second source of information about associations and dissociations of cognitive mechanisms, complementing covariations across individuals that have been instrumental for studying the relation of WM and attention for several decades (Keye, Wilhelm, Oberauer, & van Ravenzwaaij, 2009; McVay & Kane, 2012; Unsworth, Redick, Lakey, & Young, 2010; Unsworth & Robison, 2017). Measuring multiple indicators of sustained attention, WM, and other constructs to assess their covariation matrix over time, we could use structural-equation modelling to determine these constructs on the latent-variable level.
